# Genotyping of *Plasmodiophora brassicae* reveals the presence of distinct populations

**DOI:** 10.1186/s12864-018-4658-1

**Published:** 2018-04-16

**Authors:** Michael D. Holtz, Sheau-Fang Hwang, Stephen E. Strelkov

**Affiliations:** 1 Alberta Agriculture and Forestry, Field Crop Development Centre, Lacombe, Alberta Canada; 2 Alberta Agriculture and Forestry, Crop Diversification Centre North, Edmonton, Alberta Canada; 3 Department of Agricultural, Food and Nutritional Science, University of Alberta, Edmonton, Alberta Canada

**Keywords:** Clubroot, *Plasmodiophora brassicae*, Population structure, RAD sequencing

## Abstract

**Background:**

*Plasmodiophora brassicae* is a soilborne pathogen of the family Brassicaceae and the causal agent of clubroot disease. In Canada, *P. brassicae* is now one of the most important constraints to canola (*Brassica napus*) production, and is managed mainly by the deployment of resistant cultivars. In recent years, however, new strains of the pathogen have emerged that are capable of overcoming host resistance, posing new challenges for disease management. Despite its economic significance, molecular studies of *P. brassicae* are rare, mainly because this microorganism cannot be cultured outside of its host.

**Results:**

Restriction site-associated DNA sequencing (RADseq) was used to examine the genetic diversity within *P. brassicae* single-spore and field isolates collected from across Canada. The isolates included individuals that were either capable or incapable of causing disease on clubroot resistant canola cultivars. Over 8750 variants were identified through RADseq. Population analysis indicated that most isolates belonged to one of two distinct populations, corresponding with the ability of isolates to cause disease on resistant cultivars. Within each population, there were low levels of genetic diversity. One thousand and fifty of the genetic variants that distinguished the two populations were nonsynonymous, altering the coding sequences of genes.

**Conclusion:**

The application of RADseq revealed two distinct populations of *P. brassicae* in Canada, suggesting multiple introductions of the pathogen into the country. The genetic variation found here will be important for future research and monitoring of the pathogen.

**Electronic supplementary material:**

The online version of this article (10.1186/s12864-018-4658-1) contains supplementary material, which is available to authorized users.

## Background

Clubroot is an important disease of Brassica crops worldwide. It occurs in over 60 countries and results in a 10-15% reduction in yields [1]. The causal organism, *Plasmodiophora brassicae* Woronin, is a member of the eukaryotic super group Rhizaria [2]. It is a soilborne, obligate parasite with a complex lifecycle, the majority of which is spent intracellularly within host roots or as dormant resting spores in the soil [3].

Although reports of clubroot in Canada date back at least 100 years [4], its importance was considered relatively minor until the disease was found on canola (*Brassica napus* L., *B*. *rapa* L.) on the Canadian prairies in 2003 [5]. Canola is one of the major crops grown in this region, with the canola industry valued at approximately $15.5 billion annually [6]. As a consequence of the potential economic impact of *P. brassicae* on canola production, a considerable amount of research has been carried out in recent years to understand, manage and reduce the spread of this pathogen across the Canadian prairies. Despite these efforts, the distribution of *P. brassicae* has continued to expand [7]. Clubroot-resistant (CR) canola cultivars first became commercially available in 2009-2010, and the planting of CR canola quickly became the primary clubroot management strategy [8]. Unfortunately, the efficacy of these resistant cultivars as a clubroot management tool has not been as durable as first hoped. By 2013, severe clubroot symptoms were found on CR cultivars in some fields, with these symptoms shown to result from the emergence of new virulence phenotypes of *P. brassicae* [9]. These new strains of the pathogen are highly virulent on nearly all CR canola cultivars tested, suggesting that the utility of genetic resistance as a management tool is at risk [9].

Despite its importance, little is known about the population genetics of *P. brassicae*. Traditionally, the pathogen has been classified into pathotypes based on its virulence patterns on various host differential sets [4]. In Canada, six pathotypes have been identified using the classification system of Williams [10], including pathotypes 1, 2, 3, 5, 6, and 8 [8, 11]. All of these pathotypes, with the exception of pathotype 1, occur within the Canadian prairies [8]. The recently identified *P. brassicae* field isolates capable of overcoming the resistance in CR canola cultivars are also classified as pathotype 5, but this designation does not reflect their increased virulence on these hosts [9]. Infected host plants may contain multiple *P. brassicae* genotypes [12], collectively referred to as a field isolate. Single-spore isolates are sometimes obtained to produce genetically uniform samples, enabling more detailed analysis of the pathogen and avoiding the potential complications of working with heterogenetic field isolates [12].

Although molecular methods have been used with great success to study other plant pathogens, their successful application with *P. brassicae* has been difficult [13]. Since the pathogen cannot be cultured, DNA extractions are contaminated by the host DNA which can interfere with traditional molecular marker techniques such as RAPD or AFLP analysis [13, 14]. Recent advances in genomics, however, have made examination of *P. brassicae* more feasible, and its genome was recently sequenced [15, 16], as were the genomes of its hosts *B. rapa* and *B. napus* [17, 18].

The development of restriction site-associated sequencing (RADseq) has facilitated the rapid and cost effective identification of large numbers of polymorphisms within species [19]. RADseq is a form of reduced-representation library sequencing. It involves the digestion of DNA with one or more restriction enzymes followed by sequencing of the resulting fragments in a high throughput DNA sequencer. This method facilitates targeting of only a fraction of the genome, which allows for sequencing of a larger number of isolates and achieves a greater depth of coverage per locus for the same budget [19, 20]. Although RADseq is employed frequently on species with large genomes, including agricultural crop species, this technique can also be employed on species with smaller genomes, typically by varying the restriction enzymes used in order to reduce the amount of genome reduction [21]. Although contamination from host DNA is an issue with species such as *P. brassicae*, RADseq analysis does provide methods to mitigate that problem. RADseq data can be analyzed either de novo in the absence of a reference genome or by alignment to a reference genome [20]. By aligning to the reference genome, contaminating DNA from other species should be removed from the analysis. Additionally, for host species which are likely to contaminate DNA extractions, sequences aligning to the host genome can be removed prior to RADseq data analysis, further reducing the chances that DNA that is not from the target species will affect the analysis.

The overall objective of this study was to perform a genome-wide examination of the diversity within *P. brassicae* using a RADseq approach. Specifically, the aims were to: (i) determine if there is any differentiation between *P. brassicae* populations from Alberta that are virulent on CR canola and other *P. brassicae* populations that are not virulent on CR canola, and (ii) compare these pathogen populations with populations from other regions of Canada.

## Results

A total of 10,692,831 reads, providing an average of 509,182 reads per isolate, were obtained from the two sequencing runs. Of these, 3.7% of the reads were found to align to the host genomes *B. rapa* or *B. napus*. After variant calling and filtration, 7576 SNPs, 915 complex variants, and 264 MNPs were detected. The average depth of coverage was 29.3 reads/SNP locus. The frequency of SNPs in the genome averaged 315 SNPs/Mb. The number of SNPs per contig was significantly correlated (r^2^ = 0.85) with the size of the contig, whereas the density of SNPs on a contig was not (r^2^ = − 0.056). On average, isolates were heterozygous for 9.5% of the loci. The *P. brassicae* isolate AbotJE-ss1_aaf was an outlier, since it was heterozygous at 48% of called loci. The transition to transversion ratio was 1.71.

### Determination of population structure

**Fig. 1:**
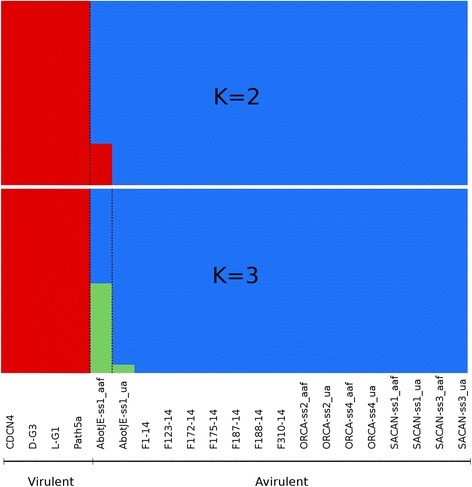
Population structure of *Plasmodiophora brassicae*. Population structure at K = 2 and K = 3 based on 7576 SNP markers. Each sample is represented by a single vertical bar and is partitioned into K coloured segments with lengths proportional to the estimated probability of membership in each of the inferred K clusters

**Fig. 2:**
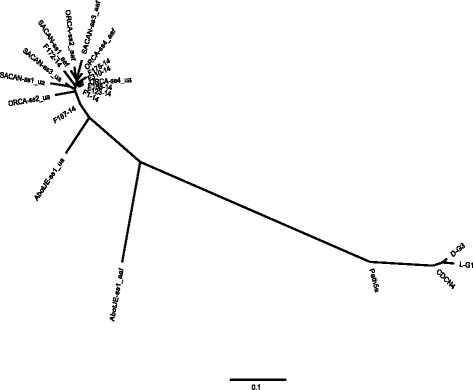
Neighbour-joining tree for 21 field and single-spore isolates of *Plasmodiophora brassicae*. Provesti’s distance was calculated from 7576 SNP markers. The field isolates CDCN4, D-G3, L-G1, and Path5a were virulent on clubroot resistant canola

Analysis of clustering implemented in fastSTRUCTURE identified an optimal K of 2 or 3, with K = 2 being the model components that best explained the structure in the data and K = 3 maximizing marginal likelihood (Fig. 1). With both values of K, the isolates that were virulent on CR canola clearly differentiated from those isolates that were avirulent on CR canola. The exception was the isolate AbotJE-ss1_aaf, which appeared admixed between the two populations when K = 2, or appeared to be admixed with the avirulent isolates and a third population when K = 3. The neighbour joining analysis supported the results of fastSTRUCTURE, with the isolates avirulent on CR canola closely related to each other but highly divergent from the virulent isolates (Fig. 2). Again, the isolate AbotJE-ss1_aaf clustered in between the two populations. The average distance between isolates virulent or avirulent on CR canola was 0.74. The average distances among virulent and avirulent isolates were only 0.035 and 0.049, respectively.

The Bayesian Information Criterion (BIC) used for Discriminant Analysis of Principal Components (DAPC) did not clearly identify a single optimum value of K, although the optimum value appeared to be in the range of K = 2 - 4 (Additional file 1). A K of 2 produced results similar to those of fastSTRUCTURE, with all isolates virulent on CR canola in one population and all isolates avirulent on CR canola clustered in a second population. Increasing K to 3 resulted in isolate AbotJE-ss1_aaf being placed in its own cluster, and when K was increased to 4, isolate Path5a also formed its own cluster (Additional file 1).

### Genetic diversity and relatedness

The nucleotide diversity (π) in all *P. brassicae* isolates examined was 0.33. Within the population that was avirulent on CR canola, π was 0.13, while it was 0.14 in the virulent population. The F_ST_ value between the virulent and avirulent populations was very high at 0.81. The measure of linkage disequilibrium (LD), rBarD, was 0.0787 (*P* < 0.001) for the avirulent population and 0.0376 (*P* = 0.080) for the virulent population.

### Variant effect prediction

**Fig. 3:**
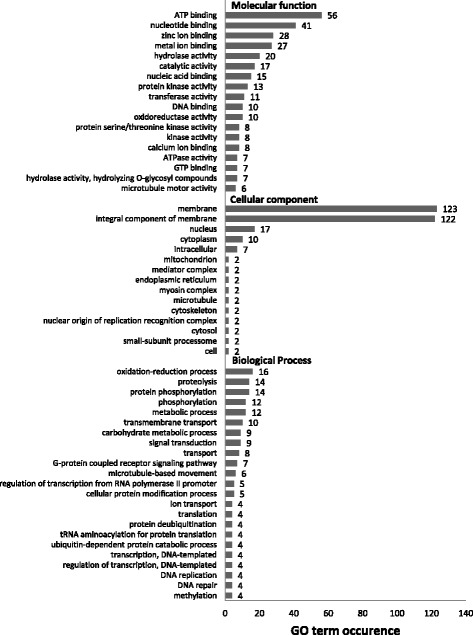
The most abundant Gene Ontology (GO) categories for proteins coded by genes with nonsynonymous variants that differentiated the isolates of *Plasmodiophora brassicae* that were virulent or avirulent on clubroot resistant canola

Of the variants distinguishing the virulent from the avirulent isolates, 44.5% of the variants occurred in coding regions. The majority (57.1%) of these were synonymous variants, and 38.2% were missense variants. The remainder had more severe coding consequences (Table 1). The 1050 nonsynonymous variants occurred in 569 genes. Three hundred and twenty nine (57.8%) of these genes were associated with 336 Gene Ontology (GO) terms in one or more of three functional categories: molecular function (148), cellular component (48), and biological process (140) (Fig. 3, Additional file 2). The remaining 240 genes (42.3%) were unannotated (Additional file 3). Among the genes with a molecular function, the most common was ATP binding (25%). The majority of genes classified as cellular components were associated with membranes (68%). Oxidation-reduction process, proteolysis, and protein phosphorylation were the most common biological processes.

**Table 1:** Summary of the effects of variants in coding regions that were distinct between the *Plasmodiophora brassicae* populations that were virulent or avirulent on clubroot resistant canola cultivars, as determined by Variant Effect Predictor

Consequence	Count
Synonymous variant	1402
Missense variant	936
Frameshift variant	84
Inframe insertion	5
Inframe deletion	8
Stop gained	4
Stop lost	1
Coding sequence variant	12

## Discussion

This study examined the relationship between recently detected *P. brassicae* isolates virulent on CR canola and other *P. brassicae* isolates from Alberta and the rest of Canada. There were clear differences between the virulent and avirulent isolates, with the two sets of isolates forming distinct populations in multiple analyses. The distant relationship between the virulent and avirulent populations was unexpected. In Canada, as elsewhere, *P. brassicae* has been traditionally characterized by its ability to overcome the resistance of different host varieties. Based on virulence phenotyping on the differential hosts of Williams [10], the field isolates D-G3 and L-G1, which are virulent on CR canola, were classified as pathotype 5 [9]. Therefore, it was hypothesized that these isolates would have been closely related to other pathotype 5 isolates, but this was not the case. The single-spore isolate ORCA-ss4 was identified previously as pathotype 5, but was a member of the population avirulent on CR canola. Obligate plant parasites with highly similar virulence phenotypes but highly divergent genotypes have been detected previously. For instance, different lineages of the wheat rust *Puccinia striiformis* f. sp. *tritici*, identified through population genomics, have been shown to contain members with identical virulence phenotypes [22].

There is little published research on the molecular diversity of *P. brassicae* with which to compare these results. The only other report from Canada is the study of Rolfe et al. [16], who sequenced the genomes of two single-spore isolates and re-sequenced the genomes of three others. The five single-spore isolates they used, SACAN-ss1, SACAN-ss3, ORCA-ss2, ORCA-ss4, and AbotJE-ss1, were also included in this research. Similar to the current study, Rolfe et al. [16] found little differentiation between the isolates with the exception of AbotJE-ss1. Moreover, as was found in the current study, AbotJE-ss1 was more distantly related to the other single-spore isolates. Since the genome data of Rolfe et al. [16] is not yet available, no direct comparisons could be made between the studies.

Results comparing the genetic diversity of *P. brassicae* in other countries have typically relied on other marker types such as RAPD. These studies generally have shown that differences between pathogen isolates from different countries are easy to detect, while differences between isolates from within a country are not as predictable [23]. Yano et al. [24] found few differences between *P. brassicae* isolates collected in Japan, whereas Manzanares-Dauleux et al. [25] found a large degree of divergence between isolates from the northwest of France. RAPD markers are considered to be notoriously unreliable, however, and there are further complications with the *Brassica*/*P. brassicae* pathosystem, such as the need to avoid the artefacts resulting from contamination by host DNA. Recent AFLP-based studies of the diversity of *P. brassicae* in Germany have revealed the species to be very genetically diverse within that country [26]. Greater diversity of *P. brassicae* within Europe compared with Canada is not surprising. While the centre of diversity of *P. brassicae* is unknown, the *Brassica* centre of diversity is in the Mediterranean [1]. That would make it much more likely that the centre of diversity of *P. brassicae* would be near the Mediterranean or Europe, and there are historical records describing what are believed to be clubroot symptoms dating back thousands of years in Europe [4]. Additionally, the general view has been that *P. brassicae* was introduced to North America from Europe [4]. This would suggest that Canadian *P. brassicae* populations are less diverse than those of Europe.

Within the populations detected here there was little differentiation between isolates. Extremely low differentiation between isolates of plant pathogens, even when using genomic methods, has been shown to occur previously [27]. This low genetic diversity within the populations along with the presence of LD suggests that *P. brassicae* in Canada may be clonal. Although the single-spore isolates that were included in duplicate clustered together, the pairs of replicate isolates tended to be as similar to various field isolates as they were to the other member of the pair (Fig. 2). This suggests that there is a degree of noise present in the data set, a problem which can occur during RADseq [28]. When dealing with clonal species it has been found that RADseq, although very capable of distinguishing clonal populations, can introduce noise that makes it difficult to infer fine-scale population structure [29].The rBarD values for the pathogen population virulent on CR canola were nonsignificant, indicating that recombination may occur. This study was not specifically designed to look for evidence of recombination, however, since the overall sample size was quite small and most isolates were sampled from distinct geographic regions, which would have deprived them of the opportunity for recombination. Additionally, noise in the data set may have affected the rBarD test. Studies to ascertain the occurrence or frequency of recombination in *P. brassicae* should probably be focused on isolates collected on a local scale, where distinct genotypes of *P. brassicae* are known to occur. The existence of a sexual cycle in *P. brassicae* has not been conclusively demonstrated thus far [30], and even in populations which have high genetic diversity, high LD also has been found [26].

Isolate AbotJE-ss1_aaf appears to be a mixed sample due to its extremely high heterozygosity and due to the detection of admixture by fastSTRUCTURE. It is not known why AbotJE-ss1_aaf appeared to be admixed. The isolate had been purified as a single-spore isolate and AbotJE-ss1_ua did not appear admixed. It is possible the isolate had been contaminated at some point during its maintenance in the greenhouse. Although AbotJE-ss1_aaf appeared to be admixed between the virulent and avirulent populations, there was also evidence that at least one additional genotype was present in the sample. There were 178 private alleles specific to the isolate and all loci that were called as triallelic were due to the presence of unique variants in AbotJE-ss1_aaf. This indicates that at least one other genotype was present in the sample. Furthermore, fastSTRUCTURE indicated that AbotJE-ss1_aaf may have been admixed with a third population when K = 3, and DAPC placed AbotJE-ss1_aaf into its own cluster when K was 3 or 4. DAPC does not assume any genetic model, making it more applicable to clonal organisms, unlike Bayesian methods such as fastSTRUCTURE [31]. The disadvantage of DAPC, however, is that it is unable to identify admixed individuals [32]. The assignment of AbotJE-ss1_aaf to its own cluster by DAPC likely reflects the inability of this method to display admixture. It is not clear as to what unique genotype was present within AbotJE-ss1_aaf, or where it came from. The presence of private alleles, absent from all other isolates analyzed, indicates that the single-spore isolate was contaminated at some point prior to DNA extraction. The number of isolates included in this study was relatively low, so it is likely that there are other distinct *P. brassicae* genotypes present in Canada that were not captured in the current analysis. It was expected that admixed samples would occur, but they would be field isolates. Other experiments based on phenotyping or RAPD markers have shown that the presence of multiple genotypes within field isolates is quite typical [12, 25]. It may be that some of the field isolates were composed of mixtures of unique genotypes as well, but this may have gone undetected due to the close relatedness between different genotypes.

For the purposes of variant calling and analysis, *P. brassicae* was treated as a diploid organism. This was done mostly to allow for the detection of isolates that contained multiple individuals by the presence of heterozygotic sites. The ploidy of *P. brassicae* varies throughout its lifecycle [33]. The resting spores that are formed within the root tissue, and which likely contributed to the majority of the DNA analysed in the study, are haploid. In contrast, the plasmodia in the roots from which the resting spores are formed are diploid [33].

In previous studies on the genetic diversity of *P. brassicae*, there were always concerns that the DNA of the host would affect the results of the analysis [25, 26, 34]. The RADseq method employed here eliminates this difficulty in two ways. First, by removing DNA sequences that align with the host genome, contaminating host DNA can be removed. Second, by aligning against the reference genome, any remaining host DNA as well as the DNA of any endophytes that may have been present should also be removed from the analysis. Additionally, the actual amount of reads that could be aligned to the host was quite low, indicating that the spore purification method employed was quite effective.

Effect prediction and GO analysis relied on the current *P. brassicae* genome, which is still quite new [15]. Currently, 57.9% of the predicted genes within the *P. brassicae* genome are annotated. Despite this relatively low percentage, numerous variants found here have a potential functional role (Additional file 1). Numerous GO terms for molecular function and biological processes were associated with variants that distinguished the virulent and avirulent isolates from each other. The proteins with cellular component GOs were dominated by membrane-related terms. The membrane is the main site of interaction between an obligate parasite, like *P. brassicae*, and its host. It is possible that some of these genes are involved in pathogenesis and the host-specific plant-pathogen interactions that distinguish different pathotypes, but additional research would be required to confirm if any of these genes are indeed pathogenesis-related. The RADseq method used here utilizes methylation sensitive restriction enzymes for the purpose of targeting gene-rich regions of fungal genomes [21]. Although the occurrence of methylation in the *P. brassicae* genome is unknown, similarity with that of many fungal and plant genomes may explain why a large number of variants were found in genes.

## Conclusion

The current results demonstrate the first successful application of reduced representation sequencing with a member of the Rhizaria. Two distinct, highly divergent, *P. brassicae* populations were detected within Canada: the established population that is avirulent on CR canola cultivars, and the recently detected population virulent on these hosts. This finding suggests that the virulent population could be a recent introduction to Alberta, or was present at a low frequency and went undetected until it was increased by the selection pressure imposed by the planting of CR canola. Each population appears to possess low levels of genetic diversity and is potentially clonal. Additional sampling including a larger number of isolates that originate from close geographic proximity to each other would help determine if additional populations exist and if there is gene flow between them. A large number of potential candidate effectors and genetic variants were identified that can be the basis of future research. The genetic variants detected here provide an important resource for the study and monitoring of *P. brassicae*. Such further monitoring has important consequences for clubroot management and resistance breeding.

## Methods

### Isolates and DNA extraction

**Fig. 4:**
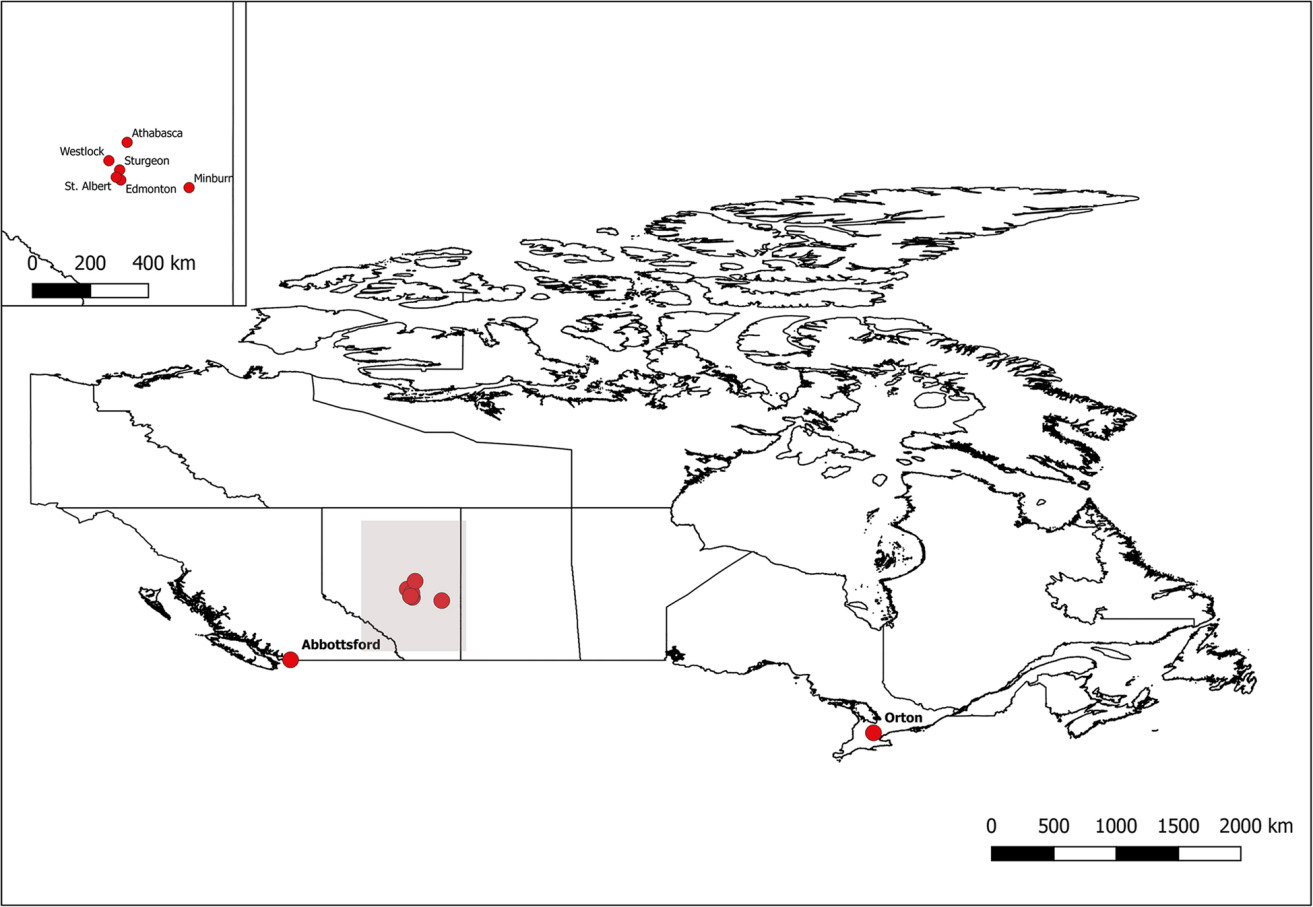
Map of Canada showing the approximate geographic origin of the *Plasmodiophora brassicae* isolates included in this study. Geographic shape file from Boundary Files, 2011 Census. Statistics Canada Catalogue no. 92-160-X. Reproduced and distributed on an “as is” basis with the permission of Statistics Canada

Twenty-one *P. brassicae* single-spore and field isolates were analysed (Table 2). These included 11 field isolates that were collected in 2013 or 2014 [9] and five isolates that were originally collected from 2003 to 2006 [11] and then purified through single-spore isolation in 2008 [12]. All field isolates and two of the single-spore isolates were from fields located within 250 km of each other in the Edmonton, Alberta, region; the remaining single-spore isolates were from Abbottsford, British Columbia, approx. 790 km west of Edmonton, and Orton, Ontario, about 2640 km east of Edmonton (Table 2, Fig. 4). For the five single-spore isolates, two different sources were used for each isolate, one that has been maintained at the University of Alberta (suffixed with ‘ua’) and one that has been maintained at Alberta Agriculture and Forestry (suffixed with ‘aaf’). The single-spore isolates were maintained on infected root material (*B. rapa* L. var. *pekinensis*, European Clubroot Differential (ECD) 05) under greenhouse conditions. Resting spores of *P. brassicae* were purified from the host tissue according to the sucrose gradient centrifugation method of Castlebury et al. [35]. The resulting purified spore suspension was centrifuged at 8000 g for 4 min to pellet the spores. DNA was then extracted from the spore pellet using an E.Z.N.A. Soil DNA Kit (Omega Bio-Tek, GA, USA). The concentrations of the DNA extractions were determined using a fluorescence-based Qubit fluorometer (Invitrogen, CA, USA) and were normalized to a concentration of 10 ng/μl.

**Table 2:** Field and single-spore isolates of *Plasmodiophora brassicae* used for restriction site associated sequencing

Isolate Name	Year	Location	Patho-type	Viru-lent^a^	Host^b^	Purity^c^	Reference
AbotJE-ss1_aaf	2006	Abbottsford, BC	6	No	*B. rapa* subsp. *pekinensis*	ssi	[10, 11]
AbotJE-ss1_ua	2006	Abbottsford, BC	6	No	*B*. *rapa* subsp. *pekinensis*	ssi	[10, 11]
CDCN4	2006	Edmonton, AB	3	Yes	*B. napus* ‘L135c’	fi	This study
D-G3	2013	Westlock, AB	5	Yes	*B*. *napus* ‘D3152’	fi	[9]
F1-14	2014	Edmonton, AB	3	No	*B*. *napus* ‘74-54RR’	fi	This study
F123-14	2014	Minburn, AB		No	*B*. *rapa* subsp. *pekinensis*	fi	This study
F172-14	2014	Athabasca, AB		No	*B*. *napus* ‘74-54RR’	fi	This study
F175-14	2014	Athabasca, AB	6	No	*B*. *napus* ‘74-54RR’	fi	This study
F187-14	2014	Sturgeon County, AB	8	No	*B*. *napus* ‘45H29’	fi	This study
F188-14	2014	Sturgeon County, AB	8	No	*B*. *napus* ‘L135c’	fi	This study
F310-14	2014	Sturgeon County, AB		No	*B*. *rapa* subsp. *pekinensis*	fi	This study
L-G1	2013	Westlock, AB	5	Yes	*B*. *napus* ‘L135c’	fi	[9]
ORCA-ss2_aaf	2006	Orton, ON	8	No	*B*. *rapa* subsp. *pekinensis*	ssi	[10, 11]
ORCA-ss2_ua	2006	Orton, ON	8	No	*B*. *rapa* subsp. *pekinensis*	ssi	[10, 11]
ORCA-ss4_aaf	2006	Orton, ON	5	No	*B*. *rapa* subsp. *pekinensis*	ssi	[10, 11]
ORCA-ss4_ua	2006	Orton, ON	5	No	*B*. *rapa* subsp. *pekinensis*	ssi	[10, 11]
Path 5a	2013	Westlock, AB	5	Yes	*B*. *napus* ‘45H26’	fi	This study
SACAN-ss1_aaf	2006	St. Albert, AB	3	No	*B*. *rapa* subsp. *pekinensis*	ssi	[10, 11]
SACAN-ss1_ua	2006	St. Albert, AB	3	No	*B*. *rapa* subsp. *pekinensis*	ssi	[10, 11]
SACAN-ss3_aaf	2006	St. Albert, AB	2	No	*B*. *rapa* subsp. *pekinensis*	ssi	[10, 11]
SACAN-ss3_ua	2006	St. Albert, AB	2	No	*B*. *rapa* subsp. *pekinensis*	ssi	[10, 11]

^a^‘Virulent’ refers to whether or not an isolate is highly virulent on Canadian clubroot resistant canola cultivars

^b^‘Host’ refers to the root material from which resting spores of *P. brassicae* were purified for the present analysis

^c^Samples were either field isolates (fi) or had been purified previously as single-spore isolates (ssi)

### Sequencing

The *P. brassicae* isolates were sequenced using RADseq as described by LeBoldus et al. [21]. Briefly, 200 ng of DNA was digested with the restriction enzyme *Hha*I, followed by digestion with *Ape*KI. The digested DNA was then purified with a Wizard PCR purification kit (Promega, WI, USA). Adapters were then ligated to the fragment end. The adapters included a unique barcode sequence and the Ion Torrent sequencing primer sequences. The adapter ligated DNA samples were combined and then purified again and size selection was performed for approximately 275 bp fragments using a SizeSelect E-Gel (Invitrogen). The size-selected DNA fragments were amplified under the following conditions: 95 °C for 60 s followed by 10 cycles of 94 °C for 30 s, 62 °C for 30 s, and 72 °C for 30 s, with a final extension at 72 °C for 300 s. The library concentration was determined by qPCR using a library quantification kit (Kapa BioSystems, Cape Town, South Africa) on a StepOnePlus Real-Time PCR System (Life Technologies, CA, USA). Two libraries were created. Sequencing was performed on an Ion Torrent PGM (Life Technologies, CA, USA) using an Ion PGM Template OT2 200 kit and a Ion PGM Hi-Q sequencing kit for 200 bp reads. Two sequencing runs were conducted, one for each library, using Ion 318 sequencing chips.

### Data processing and variant calling

Initial data processing was performed using the Ion Torrent Suite v4.4.3 (Life Technologies), which removed low quality sequences and removed the ion torrent adapters and low quality 3′ ends. Reads were demultiplexed based on the barcode sequence using Sabre (https://github.com/najoshi/sabre). As the *P. brassicae* DNA would likely be contaminated with some host DNA, the amount of host contamination was determined and removed by alignment against the *B. rapa* (assembly GCA_000309985.1) and *B. napus* (assembly GCA_000686985.1) genomes using DeconSeq 0.4.3 [36]. The demultiplexed reads were aligned to the *P. brassicae* genome (assembly GCA_001049375.1) using MOSAIK 2.2.3 [37]. Variant calling was performed using Freebayes v0.9.21 [38]. The resulting vcf file was filtered by vcflib [https://github.com/ekg/vcflib] to retain variants present in 18 out of 21 samples, with a minimum depth of 10, and a minimum Phred quality score of 20. The variant set was then filtered to include only SNPs for many analyses.

### Determination of population structure

The presence of populations (K) within *P. brassicae* was examined using model based clustering analysis implemented in fastSTRUCTURE [31]. The analysis was performed on the filtered data set containing only SNPs. The analysis was conducted using the simple prior and the default convergence criterion value of 10e-6. Ten independent runs were performed with K ranging from 1 to 10. A neighbour-joining tree based on Provesti’s distance was created to visualize relations between samples using the R packages Poppr [39] and Ape [40].

To complement the above techniques, a DAPC was used [41] to identify the number of clusters within the SNP data set. DAPC was performed using the R package adegenet [42]. The optimal number of clusters was predicted using the k-means clustering algorithm, and the BIC was then calculated with K ranging from 1 to 12. DAPC was used to assign individuals into populations.

### Population genetic analysis

The nucleotide diversity (π) and the F_ST_ between the detected populations were calculated using VCFtools v0.1.13 [43]. Multilocus linkage disequilibrium (LD) within populations was determined in adegenet using the standardized index of association (rBarD) with 1000 randomisations of the data [44].

### Variant effect prediction

Differences in variant effects between isolates virulent or avirulent on CR canola were examined using the variant effect predictor (VEP) tool [45]. First, the variant data set was filtered to include only variants that separated the virulent isolates from the avirulent isolates using SnpSift [46]. The resulting data set was then subjected to variant effect prediction using VEP. The isolate AbotJE-ss1_aaf was excluded from the analysis as it appeared to be an admixed sample. For genes that had variants causing non-synonymous coding consequences, gene ontology (GO) terms were obtained from the reference genome [15].

## Additional files

Additional file 1: Clustering of *Plasmodiophora brassicae* samples based on discriminant analysis of principal components (DAPC). (DOC 143 kb)

Additional file 2: Genes containing genetic variants that caused nonsynonymous mutations and distinguished *Plasmodiophora brassicae* isolates that were virulent on clubroot resistant canola from isolates that were avirulent on clubroot resistant canola, and the associated gene ontology terms. (TSV 113 kb)

Additional file 3: Genes containing genetic variants that caused nonsynonymous mutations and distinguished *Plasmodiophora brassicae* isolates that were virulent on clubroot resistant canola from isolates that were avirulent on clubroot resistant canola, and which lack gene ontology annotation. (TXT 3 kb)

## Abbreviations

*B. napus*: *Brassica napus*
*B. rapa*: *Brassica rapa*
BIC: Bayesian Information criterion
CR: Clubroot-resistant
DAPC: Discriminant analysis of principal components
ECD: European Clubroot Differential
GO: Gene ontology
LD: Linkage disequilibrium
*P. brassicae*: *Plasmodiophora brassicae*
RADseq: Restriction site-associated DNA sequencing
VEP: Variant effect predictor

## References

[1] Dixon GR (2009). The occurrence and economic impact of *Plasmodiophora brassicae* and clubroot disease. J Plant Growth Regul.

[2] Burki F, Kudryavtsev A, Matz MV, Aglyamova GV, Bulman S, Fiers M (2010). Evolution of Rhizaria: new insights from phylogenomic analysis of uncultivated protists. BMC Evol Biol.

[3] Hwang SF, Strelkov SE, Feng J, Gossen BD, Howard RJ. *Plasmodiophora brassicae*: a review of an emerging pathogen of the Canadian canola (*Brassica napus*) crop. Mol Plant Pathol. 2012;13(2):105-13. 10.1111/j.1364-3703.2011.00729.x PMC6638701 21726396

[4] Howard RJ, Strelkov SE, Harding MW (2010). Clubroot of cruciferous crops–new perspectives on an old disease. Can J Plant Pathol.

[5] Tewari JP, Strelkov SE, Orchard D, Hartman M, Lange RM, Turkington TK (2005). Identification of clubroot of crucifers on canola (*Brassica napus*) in Alberta. Can J Plant Pathol.

[6] Rempel CB, Hutton SN, Jurke CJ (2014). Clubroot and the importance of canola in Canada. Can J Plant Pathol.

[7] Gossen BD, Strelkov SE, Manolii VP, Rennie DC, Cao T, Hwang SF (2015). Spread of *Plasmodiophora brassicae* on canola in Canada, 2003–2014: old pathogen, new home. Can J Plant Pathol.

[8] Strelkov SE, Hwang SF (2014). Clubroot in the Canadian canola crop: 10 years into the outbreak. Can J Plant Pathol.

[9] Strelkov SE, Hwang SF, Manolii VP, Cao T, Feindel D (2016). Emergence of new virulence phenotypes of *Plasmodiophora brassicae* on canola (*Brassica napus*) in Alberta, Canada. Eur J Plant Pathol.

[10] Williams PH (1966). A system for the determination of races of *Plasmodiophora brassicae* that infect cabbage and rutabaga. Phytopathology.

[11] Strelkov SE, Tewari JP, Smith-Degenhardt E (2006). Characterization of *Plasmodiophora brassicae* populations from Alberta, Canada. Can J Plant Pathol.

[12] Xue S, Cao T, Howard RJ, Hwang SF, Strelkov SE (2008). Isolation and variation in virulence of single-spore isolates of *Plasmodiophora brassicae* from Canada. Plant Dis.

[13] Siemens J, Bulman S, Rehn F, Sundelin T (2009). Molecular biology of *Plasmodiophora brassicae*. J Plant Growth Regul.

[14] Heo SH, Jang SJ, Choi JS, Jang CS, Song JY, Kim HG (2009). Chinese cabbage clubroot pathogen, *Plasmodiophora brassicae*, is genetically stable. Mycobiology.

[15] Schwelm A, Fogelqvist J, Knaust A, Jülke S, Lilja T, Bonilla-Rosso G (2015). The *Plasmodiophora brassicae* genome reveals insights in its life cycle and ancestry of chitin synthases. Sci Rep.

[16] Rolfe SA, Strelkov SE, Links MG, Clarke WE, Robinson SJ, Djavaheri M, Malinowski R, Haddadi P, Kagale S, Parkin IAP, Taheri A, Borhan MH (2016). The compact genome of the plant pathogen *Plasmodiophora brassicae* is adapted to intracellular interactions with host *Brassica* spp. BMC Genomics.

[17] Wang X, Wang H, Wang J, Sun R, Wu J, Liu S (2011). The genome of the mesopolyploid crop species *Brassica rapa*. Nat Genet.

[18] Chalhoub B, Denoeud F, Liu S, Parkin IA, Tang H, Wang X (2014). Early allopolyploid evolution in the post-Neolithic *Brassica napus* oilseed genome. Science.

[19] Davey JW, Blaxter ML (2011). RADSeq: next-generation population genetics. Brief Funct Genomics.

[20] Andrews KR, Good JM, Miller MR, Luikart G, Hohenlohe PA (2016). Harnessing the power of RADseq for ecological and evolutionary genomics. Nat Rev Genet.

[21] LeBoldus JM, Kinzer K, Richards J, Ya Z, Yan C, Friesen TL, Brueggeman R (2014). Genotype-by-sequencing of the plant-pathogenic fungi *Pyrenophora teres* and *Sphaerulina musiva* utilizing ion torrent sequence technology. Mol Plant Pathol.

[22] Hubbard A, Lewis CM, Yoshida K, Ramirez-Gonzalez RH, de Vallavieille-Pope C, Thomas J, Kamoun S, Bayles R, Uauy C (2015). Field pathogenomics reveals the emergence of a diverse wheat yellow rust population. Genome Biol.

[23] Buhariwalla H, Greaves S, Magrath R, Mithen R (1995). Development of specific PCR primers for the amplification of polymorphic DNA from the obligate root pathogen *Plasmodiophora brassicae*. Physiol Mol Plant P.

[24] Yano S, Tanaka S, Ito S, Kameya-Iwaki M (1997). Variations of random amplified polymorphic DNA (RAPD) patterns among field populations of *Plasmodiophora brassicae*. Ann Phytopathol Soc Jpn.

[25] Manzanares-Dauleux MJ, Divaret I, Baron F, Thomas G (2001). Assessment of biological and molecular variability between and within field isolates of *Plasmodiophora brassicae*. Plant Pathol.

[26] Strehlow B, de Mol F, Struck C (2014). History of oilseed rape cropping and geographic origin influence the genetic structure of *Plasmodiophora brassicae* populations. Phytopathology.

[27] Milgroom MG, del Mar J-GM, García CO, Drott MT, Jiménez-Díaz RM (2014). Recombination between clonal lineages of the asexual fungus *Verticillium dahliae* detected by genotyping by sequencing. PLoS One.

[28] Davey JW, Cezard T, Fuentes-Utrilla P, Eland C, Gharbi K, Blaxter ML (2013). Special features of RAD sequencing data: implications for genotyping. Mol Ecol.

[29] Mesak F, Tatarenkov A, Earley RL, Avise JC (2014). Hundreds of SNPs vs. dozens of SSRs: which dataset better characterizes natural clonal lineages in a self-fertilizing fish?. Front Ecol Evol.

[30] Donald EC, Porter IJ (2014). Clubroot in Australia: the history and impact of *Plasmodiophora brassicae* in *Brassica* crops and research efforts directed towards its control. Can J Plant Pathol.

[31] Raj A, Stephens M, Pritchard JK (2014). fastSTRUCTURE: Variational inference of population structure in large SNP datasets. Genetics.

[32] Grünwald NJ, Goss EM (2011). Evolution and population genetics of exotic and re-emerging pathogens: novel tools and approaches. Annu Rev Phytopathol.

[33] Kageyama K, Asano T (2009). Life cycle of *Plasmodiophora brassicae*. J Plant Growth Regul.

[34] Klewer A, Luerben H, Graf H, Siemens J (2001). Restriction fragment length polymorphism markers to characterize *Plasmodiophora brassicae* single-spore isolates with different virulence patterns. J Phytopathol.

[35] Castlebury LA, Maddox JV, Glawe DA (1994). A technique for the extraction and purification of viable *Plasmodiophora brassicae* resting spores from host root tissue. Mycologia.

[36] Schmieder R, Edwards R (2011). Fast identification and removal of sequence contamination from genomic and metagenomic datasets. PLoS One.

[37] Lee WP, Stromberg MP, Ward A, Stewart C, Garrison EP, Marth G (2014). MOSAIK: a hash-based algorithm for accurate next-generation sequencing short-read mapping. PLoS One.

[38] Garrison E, Marth G (2012). Haplotype-based variant detection from short-read sequencing. arXiv Preprint arXiv.

[39] Kamvar ZN, Tabima JF, Grünwald NJ (2014). Poppr: an R package for genetic analysis of populations with clonal, partially clonal, and/or sexual reproduction. Peer J.

[40] Paradis E, Claude J, Strimmer K (2004). APE: analysis of phylogenetics and evolution in R language. Bioinformatics.

[41] Jombart T, Devillard S, Balloux F (2010). Discriminant analysis of principal components: a new method for the analysis of genetically structured populations. BMC Genet.

[42] Jombart T (2008). Adegenet: a R package for the multivariate analysis of genetic markers. Bioinformatics.

[43] Danecek P, Auton A, Abecasis G, Albers CA, Banks E, DePristo MA (2011). The variant call format and VCFtools. Bioinformatics.

[44] Agapow PM, Burt A (2001). Indices of multilocus linkage disequilibrium. Mol Ecol Notes.

[45] McLaren W, Gil L, Hunt SE, Riat HS, Ritchie GR, Thormann A (2016). The ensembl variant effect predictor. Genome Biol.

[46] Cingolani P, Patel VM, Coon M, Nguyen T, Land SJ, Ruden DM, Lu X (2012). Using *Drosophila melanogaster* as a model for genotoxic chemical mutational studies with a new program, SnpSift. Toxicogenomics Non-mammalian Species. Front Genet.

