# Supplementary material for: Genotyping of Plasmodiophora brassicae reveals the presence of distinct populations
Source: BMC Genomics. 2018 Apr 16;19:254. doi: 10.1186/s12864-018-4658-1 (PMC5902848; doi:10.1186/s12864-018-4658-1)
Supplement: Supplementary file 1 — Clustering of Plasmodiophora brassicae samples based on discriminant analysis of principal components (DAPC). (DOC 143 kb) [file 12864_2018_4658_MOESM1_ESM.doc]

Additional File 1: Clustering of *Plasmodiophora brassicae* samples based on discriminant analysis of principal components (DAPC).

Application of DAPC to 7576 SNP markers in *P. brassicae*. a) The Bayesian information criteria indicated the optimal K was in the range of 2-4. b) DAPC plot for K = 3. c) DAPC plot for K = 4.


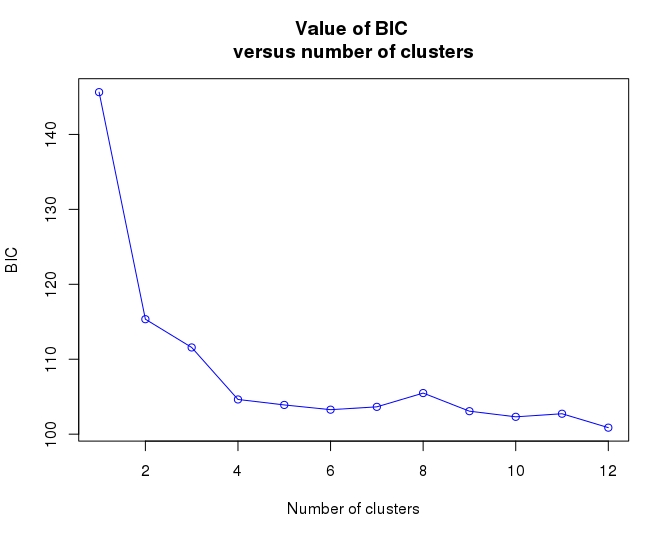


**A)**


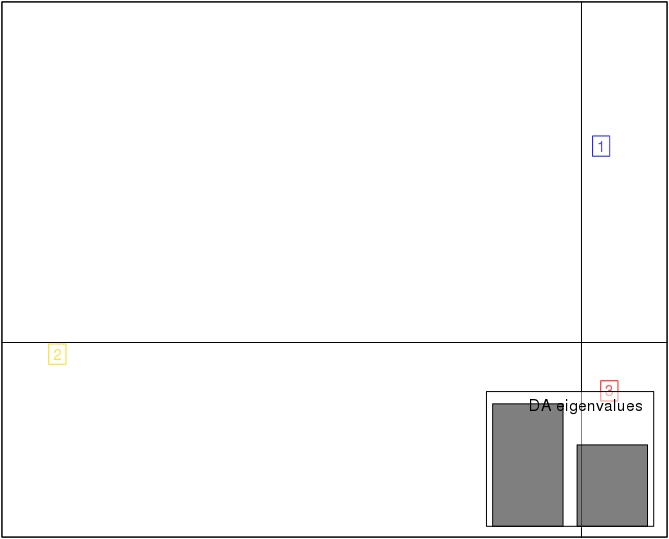


**B)**


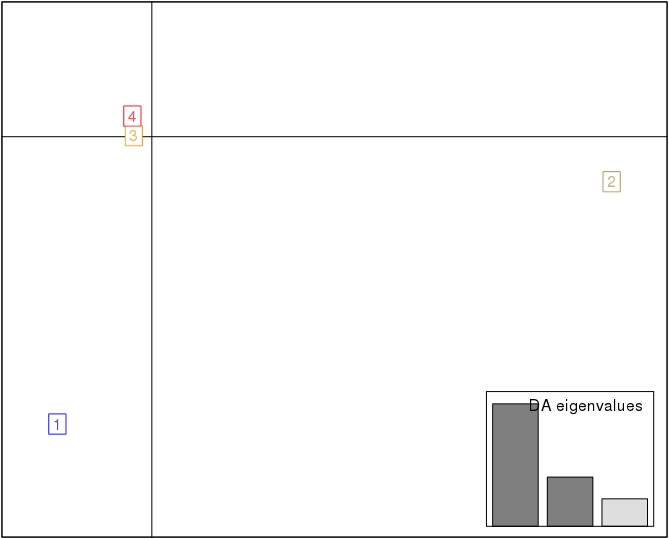


**C)**

Assignment of *Plasmodiophora* *brassicae* isolates into genetic groups by discriminate analysis of principal components, for different numbers of clusters.

| Isolate Name | DAPC group k=2 | DAPC group k=3 | DAPC group k=4 |
| --- | --- | --- | --- |
| AbotJE-ss1_aaf | 2 | 2 | 2 |
| AbotJE-ss1_ua | 2 | 3 | 3 |
| CDCN4 | 1 | 1 | 4 |
| D-G3 | 1 | 1 | 4 |
| F1-14 | 2 | 3 | 3 |
| F123-14 | 2 | 3 | 3 |
| F172-14 | 2 | 3 | 3 |
| F175-14 | 2 | 3 | 3 |
| F187-14 | 2 | 3 | 3 |
| F188-14 | 2 | 3 | 3 |
| F310-14 | 2 | 3 | 3 |
| L-G1 | 1 | 1 | 4 |
| ORCA-ss2_aaf | 2 | 3 | 3 |
| ORCA-ss2_ua | 2 | 3 | 3 |
| ORCA-ss4_aaf | 2 | 3 | 3 |
| ORCA-ss4_ua | 2 | 3 | 3 |
| Path 5a | 1 | 1 | 1 |
| SACAN-ss1_aaf | 2 | 3 | 3 |
| SACAN-ss1_ua | 2 | 3 | 3 |
| SACAN-ss3_aaf | 2 | 3 | 3 |
| SACAN-ss3_ua | 2 | 3 | 3 |
